# Southern Journal of the Medical and Physical Sciences

**Published:** 1853-04

**Authors:** 


*Southern Journal of the Medical and Physical Sciences.*

THIS journal is published every two months, and is divided into three departments. The first is devoted to practical medicine and surgery, and is under the editorial management of Drs. J. W. King, W. P. Jones and F. A. Ramsay; the second, devoted to chemistry and pharmacy, is edited by R. O. Currey, M. D., and the third, embracing dental surgery, is confided to the editorship of B. Wood, M. D., Surgeon Dentist, Thomas A. Atchison, of Kentucky, is corresponding editor.

We have received Nos. 1 and 2. The paper and typographical execution, as well as the character of the matter, are highly creditable to the publishers, contributors and editors. Presenting such strong claims upon both the medical and dental professions, the Southern Journal of Medical and Physical Sciences, ought, and no doubt will, receive a liberal patronage. We welcome it most cordially to our list of exchanges, and wish it abundant success. It is published at Nashville, Tenn.

